# Lecture on Hare-Lip, &c.

**Published:** 1839-12-21

**Authors:** William Gibson


*Saturday, December 7th*, 1839.

### LECTURE ON HARE-LIP, &c.

BY WILLIAM GIBSON, M. D.

The first case I show you to-day, gentlemen, is the woman with fractured clavicle, whom you saw at the commencement of the course. It is now three weeks and three days since the occurrence of the accident, and you observe the great change in her appearance since you last saw her. At that time the fracture was two days old, the shoulder of the left side depressed, the sternal fragment projecting, and the humeral fragment far below its natural level; the swelling and deformity were also considerable. Now, you see the shoulder raised to its proper height, the clavicle presents an almost unbroken horizontal plane, the swelling has subsided in a great measure, and by the time complete reunion has taken place, little or no difference will be perceptible between the two bones. The prominence you observed over the seat of the fracture is the callus which is being poured out to effect the bony union, and will be more or less absorbed in the course of a few weeks. The bandage of Desault has been constantly worn,—the last roller of which, you observe, I have removed, to give you a view of the injured bone. The patient, so far from complaining of this apparatus, is only comfortable when it is on,—in this instance, at least, annulling the frequent charge against it, of inconvenience and distress to females. Her mammæ, too, are by no means small, yet have never, in the least, been interfered with.

The next patient you will at once recognise: it is the little fellow I operated on, last Saturday, for hare-lip. You see that, so far, no benefit has resulted; and a moment's recurrence to the peculiarities of the case will explain the reason of it. In drawing back the lip of the right side, I expose the projection of the upper maxillary bone, from which the first two or three teeth stand out irregularly: over this projection and the teeth the lip had to be drawn to meet the lip of the opposite side, which, together with the left maxillary bone, is sunk considerably below the level of the right side. I had also to drag up the lip of the left side, to meet that of the right, so as to place the cut edges of the two in apposition.

The strain, then, upon which the parts were put, increased as it was by this central projection, resembling in its action the bridge of a violin, was the main cause of difficulty.

Another circumstance may have contributed to prevent reunion. The boy was put to bed after the operation, and passed the day comfortably, as also the day after. On Monday afternoon, however, without any apparent cause, a slight hæmorrhage took place from the upper part of the wound, but was spontaneously arrested. On Wednesday I saw him, having come out for the purpose of removing the pins, but learning that hæmorrhage had occurred, declined removing them, lest it should return, and accordingly left them untouched. At that time union was evidently taking place at the lower part of the lip, but not at the upper, from which alone the blood oozed. On Thursday afternoon additional bleeding occurred from the same part. This was also spontaneously arrested; and on Friday I removed the pins, seeing that they were about to ulcerate through, when the lips immediately separated and resumed their old position.

There is no reason to believe that the hæmorrhage spoken of was from the arteries divided during the operation. There seems to be a hæmorrhagic tendency in this boy, for I have seldom before seen so much blood issue during such an operation in a subject so young.

I shall not repeat the operation to-day, because the parts are in a state of inflammation, and pins or sutures, if applied to inflamed parts, would probably break out: I shall wait patiently, therefore, until inflammation subsides, and shall then determine whether it will be worth while to renew the attempt without previously taking away the projection of the jaw and the teeth. You probably remember that I stated that this obstacle might impede reunion,—but as the removal of it would not only be very painful, but would also sacrifice the boy's teeth, I wished to try in the first place to accomplish the closure of the fissure, if possible, without that sacrifice. I shall give the subject, therefore, due consideration, and determine what further course should be pursued.

[Dr. Gibson now introduced two cases of syphilis, and commenced a history of that disease,—promising to continue the subject at the next meeting. Both lectures will, therefore, be contained in the next number.]

